# PGE_2_ suppression of innate immunity during mucosal bacterial infection

**DOI:** 10.3389/fcimb.2013.00045

**Published:** 2013-08-21

**Authors:** Mallory Agard, Saja Asakrah, Lisa A. Morici

**Affiliations:** Department of Microbiology and Immunology, Tulane University School of MedicineNew Orleans, LA, USA

**Keywords:** bacteria, prostaglandin, COX, immunotherapeutic, mucosal, infection

## Abstract

Prostaglandin E2 (PGE2) is an important lipid mediator in inflammatory and immune responses during acute and chronic infections. Upon stimulation by various proinflammatory stimuli such as lipopolysaccharide (LPS), interleukin (IL)-1β, and tumor necrosis factor (TNF)-α, PGE2 synthesis is upregulated by the expression of cyclooxygenases. Biologically active PGE2 is then able to signal through four primary receptors to elicit a response. PGE2 is a critical molecule that regulates the activation, maturation, migration, and cytokine secretion of several immune cells, particularly those involved in innate immunity such as macrophages, neutrophils, natural killer cells, and dendritic cells. Both Gram-negative and Gram-positive bacteria can induce PGE2 synthesis to regulate immune responses during bacterial pathogenesis. This review will focus on PGE2 in innate immunity and how bacterial pathogens influence PGE2 production during enteric and pulmonary infections. The conserved ability of many bacterial pathogens to promote PGE2 responses during infection suggests a common signaling mechanism to deter protective pro-inflammatory immune responses. Inhibition of PGE2 production and signaling during infection may represent a therapeutic alternative to treat bacterial infections. Further study of the immunosuppressive effects of PGE2 on innate immunity will lead to a better understanding of potential therapeutic targets within the PGE2 pathway.

## Introduction

Prostaglandin E_2_ (PGE_2_) is an important lipid mediator in inflammatory and immune responses during acute and chronic infections (Phipps et al., 1991; Yu and Chadee, 1998; Harris et al., 2002; Nagamatsu and Schust, 2010). Upon stimulation by various proinflammatory stimuli such as lipopolysaccharide (LPS), interleukin (IL)-1β, and tumor necrosis factor (TNF)-α, PGE_2_ synthesis is upregulated by the expression of one of three cyclooxygenases (Filion et al., 2001; Kis et al., 2006; Park et al., 2006). Biologically active PGE_2_ is then able to signal through four primary receptors to elicit a response (Sugimoto et al., 1992; Honda et al., 1993; Nishigaki et al., 1996; Hata and Breyer, 2004). Molecular concentrations of PGE_2_ and receptor signaling are both influential in regulating proinflammatory and immunosuppressive immune cell phenotypes (Kalinski, 2012). PGE_2_ is a critical molecule that regulates the activation, maturation, migration, and cytokine secretion of several immune cells, particularly those involved in innate immunity such as macrophages, neutrophils, natural killer cells, and dendritic cells (Bankhurst, 1982; Goto et al., 1983; Kaliński et al., 1997; Yu and Chadee, 1998; Aronoff et al., 2004; Serezani et al., 2007; Nagamatsu and Schust, 2010). Both Gram-negative and Gram-positive bacteria can induce PGE_2_ synthesis to regulate immune responses during bacterial pathogenesis (Harris et al., 2002; Hessle et al., 2003). This review will focus on PGE_2_ in innate immunity and how bacterial pathogens influence PGE_2_ production during enteric and pulmonary infections. Inhibition of PGE_2_ production, recognition, and signaling may lead to therapeutic alternatives to regulate the innate immune response during bacterial infection. Active mechanisms utilized by bacteria may also promote PGE_2_ synthesis during pathogenesis. Examination of these mechanisms could elicit a better understanding of disease progression and infection outcome.

### PGE_2_ production

**Figure 1:**
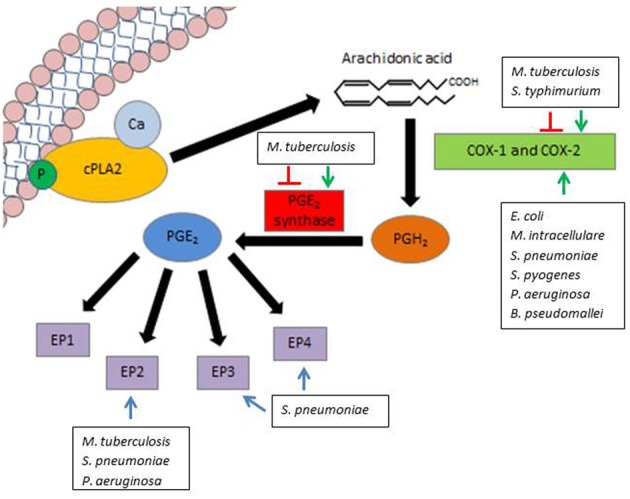
**Modulation of PGE_2_ by pathogenic bacteria.** PGE_2_ synthesis is initiated by the release of AA from the lipid cell membrane by the enzyme cPLA2. Either COX-1 (constitutively expressed) or COX-2 (inducible) can utilize AA as a substrate to produce the precursor PGH_2_. PGH_2_ is converted to biologically active PGE_2_ by means of the enzyme PGE_2_ synthase. PGE_2_ can then readily signal through one of four EP, denoted EP1, EP2, EP3, and EP4. Various pathogens influence PGE_2_ production and signaling along different steps of this pathway. Green arrows indicate activation of COX-2 or PGES, while red lines indicate inhibition of COX-2 or PGES by select pathogens. Blue lines indicate a potential role for EP signaling in pathogen survival.

While PGE_2_ can be produced by all cell types, immune cells are a primary source of PGE_2_ production during an inflammatory response (Kalinski, 2012). Within these cells, PGE_2_ is derived from the release of arachidonic acid (AA) from cell membranes by phospholipase A2 (PLA2) enzymes. While there are multiple members within the PLA2 family, the most utilized enzyme for PGE_2_ synthesis is the cytosolic calcium-dependent PLA2 (cPLA2) (Lambeau and Lazdunski, 1999). Subsequently, one of two primary cyclooxygenases utilizes AA as a substrate to produce the biological precursor prostaglandin H_2_ (PGH_2_). The two cyclooxygenases available for this reaction are COX-1 (constitutively active at basal levels) and COX-2 (highly inducible by inflammatory cytokines and growth factors) (Phipps et al., 1991). PGE_2_ is then enzymatically produced as an end product of the reaction with the aid of PGE_2_ synthase (PGES) (Park et al., 2006). Biologically active PGE_2_ can then readily signal through one of four eicosanoid receptors (EP) (Figure 1). The rate of PGE_2_ production during an immune response is primarily believed to be dependent upon the expression and activity of COX-2 (Kalinski, 2012), thus it is an important enzyme on which to focus when examining PGE_2_. PGE_2_ is relatively stable *in vitro*, yet is rapidly degraded in tissues by 15-hydroxyprostaglandin dehydrogenase (15-PGDH) (Fitzpatrick et al., 1980; Tai et al., 2002). Accordingly, in order to examine PGE_2_ under biological conditions, it is necessary to account for its rate of production via COX-2 and PGES and its degradation in response to different stimuli.

### PGE_2_ receptor signaling

There are four known PGE_2_ receptors designated EP1, EP2, EP3, and EP4, with at least three splice variants of EP3 recognized as EP3α, EP3β, and EP3γ. This diversity of PGE_2_ receptors influences the pro-inflammatory and immunosuppressive functions of this molecule within the body under different environmental conditions. EP3 and EP4 are considered to be high-affinity receptors, requiring lower levels of PGE_2_ for signaling. Conversely, EP1 and EP2 demand higher concentrations of PGE_2_ for proper signaling. Additionally, the four PGE_2_ receptors vary in their signal durations (Sugimoto et al., 1992; Honda et al., 1993; Nishigaki et al., 1996; Hata and Breyer, 2004). While the PGE_2_ receptors have fundamental differences in affinity and signal durations, there are mechanistic similarities between some of these receptors. For example, EP2 and EP4 are both G_s_-coupled receptors that signal primarily through the adenylate cyclase-dependent cAMP/PKA/CREB pathways. EP2 and EP4 are the predominant receptors responsible for the anti-inflammatory and immunosuppressive effects of PGE_2_ (Fujino et al., 2005). Both receptors are primarily thought to function in a cAMP-dependent manner, however EP4 also signals in a phosphatidylinositol 3-kinase (PI3K)-dependent manner to activate the extracellular-signal-regulated kinase 1/2 (ERK1/2) pathway (Fujino et al., 2003). Conversely, EP1 and EP3 do not require cAMP for activation. Few studies have examined the low-affinity EP1, although PGE_2_ signaling through this receptor leads to an increase in the release of cellular calcium (Hata and Breyer, 2004). Signaling through EP3 primarily involves G_i_-coupled receptors that inhibit the activity of adenylate cyclase, and consequently decrease levels of cAMP in the cell. Nevertheless, EP3 splice variants are also able to signal through G_s_-coupled receptors, enhancing the diverse signaling ranges among these PGE_2_ receptors (Sugimoto et al., 1992).

The diversity of receptors, signaling pathways, and signal duration enables PGE_2_ to act as an adaptable signaling molecule in a wide range of cell types in response to environmental stimuli. The complexities of PGE_2_ signaling help address its paradoxical ability to elicit both inflammatory and immunosuppressive responses under various concentrations and environmental conditions at early and late stages of bacterial infection (Hessle et al., 2003; Stefanelli et al., 2012). Furthermore, while PGE_2_-mediated immunoregulation is essential for maintaining homeostasis, the immunosuppressive effects of PGE_2_ during innate immune responses may be detrimental during bacterial infection, as examined in depth below.

### PGE_2_ and innate immunity

#### Neutrophils

Neutrophils are the first leukocytes recruited to sites of infection during an innate immune response. These cells possess several immune defense mechanisms including phagocytosis, proteolytic enzymes, oxygen-reactive agents, and inflammatory mediators. Accordingly, proper migration as well as signaling between these granulocytes and other immune cells is important to allow for an effective immune response at early stages of infection. Activation and aggregation of human neutrophils is inhibited after exogenous treatment with PGE_2_ *in vitro* (Ney and Schrör, 1991; Wheeldon and Vardey, 1993; Talpain et al., 1995). PGE_2_ also inhibits the activation of rat and guinea pig neutrophils *in vitro*, suggesting a conserved inhibitory role of PGE_2_ signaling among mammalian immune responses (Ham et al., 1983; Takenawa et al., 1986; Wise and Jones, 1994; Wise, 1996). Activation of mammalian neutrophils by formylmethionyl-leucyl-phenylalanine (fMLP) is inhibited by PGE_2_ in an EP2-dependent manner (Takenawa et al., 1986; Burelout et al., 2004, 2007). Inhibition of EP2 signaling improves neutrophil migration to promote bacterial clearing and enhances mouse survival following intratracheal infection with *Pseudomonas aeruginosa* (Sadikot et al., 2007; Aronoff et al., 2012). Bacterial pathogens and their structural components directly promote PGE_2_ synthesis by neutrophils during infections. For example, *Streptococcus pneumoniae* infection induces PGE_2_ production by human neutrophils and obstructs activation and migration *in vitro* (Cockeran et al., 2001). Neutrophils also produce increased concentrations of PGE_2_ after treatment with *E. coli* LPS or post-infection with *P. aeruginosa* in rat and mouse models, respectively (He et al., 2001; Alba-Loureiro et al., 2004). Since neutrophils represent a first line of defense against infection, it is important to further elucidate PGE_2_ production during bacterial infection and examine its immunomodulatory effects on the antimicrobial functions of neutrophils.

#### Macrophages

Through phagocytosis and the generation of a strong cytokine response, macrophages are important cells in innate immune responses and immunomodulation. While PGE_2_ is able to locally attract macrophages at early stages of inflammation (Nakayama et al., 2006), macrophage activation can be inhibited by PGE_2_ through EP2 signaling (Zaslona et al., 2012). The phagocytic properties of alveolar macrophages are inhibited in an EP2-dependent manner during infection with *Klebsiella pneumoniae* and *S. pneumoniae* in the rat and mouse models, respectively. Phagocytosis is restored through the inhibition of PGE_2_ synthesis with non-selective COX inhibitors such as indomethacin (Aronoff et al., 2004; Aronoff, 2012). The phagocytic properties of macrophages are dampened by PGE_2_ through the induction of immunosuppressive IL-1R-associated kinase-M (IRAK-M), impairing bacterial clearance of *P. aeruginosa* (Hubbard et al., 2010). PGE_2_ also affects the inflammatory response of macrophages during infection by altering cell signaling and inhibiting bactericidal mechanisms. Upon PGE_2_ stimulation, NAPDH oxidase is inhibited inside the macrophage, leading to reduced killing of *K. pneumoniae* (Serezani et al., 2007). PGE_2_ also suppresses macrophage activity by inhibiting the production of nitric oxide radicals (Marotta et al., 1992; Asakrah et al., 2013). PGE_2_ alters the cytokine response of macrophages and promotes an immunosuppressive phenotype. Most notable perhaps is that PGE_2_ induces the production of immunoregulatory cytokines, such as IL-10 and IL-17 (Kunkel et al., 1986, 1988; Huang et al., 1998; Stolina et al., 2000; Liu et al., 2012). Phosphatase and tensin homolog deleted on chromosome 10 (PTEN) is a downstream product of PGE_2_ signaling that negatively regulates alveolar macrophage phagocytosis and bacterial killing during *P. aeruginosa* infection. Inhibition or genetic knockout of PTEN restores the phagocytic functions of macrophages and enhances bacterial clearance *in vivo* (Hubbard et al., 2010).

#### Natural killer cells

Natural killer (NK) cells are potent granulocytes important in controlling infection during innate immune responses. While NK cells are most commonly associated with controlling viral infections, they are also important during bacterial infection. These cells respond to changes in the cytokine profile during infection in order to lyse infected cells, and PGE_2_ has a negative effect on the cytolytic activities of NK cells by suppressing their responsiveness to cytokines such IL-12 and IL-15 (Bankhurst, 1982; Goto et al., 1983; Joshi et al., 2001; Walker and Rotondo, 2004). In a leukemia rat model, an increase in PGE_2_ concentration is associated with diminished NK cell cytolysis and decreased animal survival, which is relieved upon COX inhibition by etodolac (Inbar et al., 2011). NK cells also secrete IFN-γ as a signaling mechanism to activate macrophages during the innate immune response and to aid dendritic cells in driving Th1 responses. PGE_2_ suppresses NK cell-mediated activation of macrophages by inhibiting the production of IFN-γ (Mailliard et al., 2005). Not only does PGE_2_ have an inhibitory effect on the cytokine response of NK cells, but it also downregulates the expression of receptors important for NK cell effector functions, including CD94/NKG2C, DNAM-1, NKp80, 2B4, and CD161. PGE_2_ also has a deleterious effect on the homing, migration, and survival of NK cells in humans infected with Human Herpes Virus 8 who have developed Kaposi's sarcoma (Dupuy et al., 2012). This demonstrates similarity to PGE_2_'s ability to negatively affect the aggregation of neutrophils, suggesting that there may be a conserved signaling mechanism across immune cell types. Further research must be conducted in order to elucidate the effect of PGE_2_ on NK cell activity during bacterial infection.

#### Dendritic cells

Dendritic cells (DCs) process and present antigen to immune cells during innate and adaptive immune responses and are consequently important in controlling disease progression and outcome. They initiate an adaptive immune response and are key participants in shifting immunity between Th1 and Th2 responses. PGE_2_ can disrupt DC differentiation at early stages of development (Kaliński et al., 1997). At later stages of DC differentiation, PGE_2_ can hasten DC maturation in the presence of IL-1β and TNF-α (Rieser et al., 1997; Kaliński et al., 1998). DCs that are fully developed, but functionally immature are prompted by PGE_2_ to migrate to lymph nodes (Jonuleit et al., 1997). Enhanced DC migration may be due to the ability of PGE_2_ to induce the expression of the lymphoid-homing chemokine receptor CCR7 on DCs (Luft et al., 2002; Scandella et al., 2002; Kalinski, 2012). However, PGE_2_ suppresses the production of chemokines, such as the CCR7 ligand CCL19, by DCs and inhibits naïve T cell attraction in the lymph nodes (Muthuswamy et al., 2010). PGE_2_ also impairs the ability of DCs to induce NK cell-mediated immunity (Gustafsson et al., 2008). These contrasting DC characteristics may be in part due to differences in PGE_2_ concentrations as well as receptor signaling. While it is generally accepted that DCs matured in the presence of PGE_2_ promote T cell expansion, these particular DCs suppress Th1 responses and support Th2 responses (Kalinski, 2012). For example, while PGE_2_-matured DCs effectively prime naïve T cells (Jonuleit et al., 1997), they also suppress the cytotoxic T lymphocyte (CTL) response (Obermajer et al., 2011). This alteration may be in part due to a transformed cytokine profile of PGE_2_-matured DCs. In particular, DCs matured in the presence of PGE_2_ display an enhanced production of immunosuppressive cytokines such as IL-10 (Kaliński et al., 1997) and suppress their own production of proinflammatory cytokines such as IL-12p70 (Kaliński et al., 1998). By shifting cytokine profiles away from a Th1 and toward a Th2 response, PGE_2_ may in fact promote the maturation of DCs that are better-suited to allow for intracellular bacteria to establish infection.

## The role of PGE_2_ during bacterial infection

Multiple bacterial pathogens elicit an increase in PGE_2_ production upon infection. Both Gram-negative and Gram-positive bacteria are able to induce PGE_2_ synthesis, yet Gram-negative bacteria elicit a stronger PGE_2_ response by human monocytes (Hessle et al., 2003). While passive recognition of LPS can contribute to PGE_2_ production in response to Gram-negative bacteria (Alba-Loureiro et al., 2004), it is becoming apparent that bacteria also actively induce PGE_2_ production during infection. Levels of PGE_2_ are highly regulated in the lung and gastrointestinal tract to maintain the integrity of the mucosal barrier (Takeuchi et al., 2010; Bozyk and Moore, 2011), and bacteria may modulate PGE_2_ biosynthesis to aid colonization of the lung and gut. In fact, enhanced PGE_2_ synthesis by immune cells appears to be a conserved event during bacterial infections within the mucosa, and this will be discussed in the following sections.

### Enteric infections

#### Salmonella

*Salmonella* is a Gram-negative facultative intracellular bacillus that is able to infect and survive inside several cell types including intestinal epithelial cells and macrophages. Several serotypes of *Salmonella* including *S. enterica*, *S. dublin*, and *S. typhimurium* induce the expression on PGE_2_ during infection (Ochman et al., 1996; Eckmann et al., 1997; Uchiya and Nikai, 2004). One of *Salmonella*'s most well-characterized virulence factors is the pathogenicity island 2 (SPI-2). This pathogenicity island is necessary for growth within the macrophage and is an important virulence factor in establishing infection in mice (Ochman et al., 1996; Cirillo et al., 1998; Hensel et al., 1998). SpiC, an important gene product encoded within SPI-2, is necessary for survival of *S. typhimurium* within macrophages (Uchiya et al., 1999). SpiC activates the ERK1/2 signal transduction pathway to enhance COX-2 expression and PGE_2_ synthesis in infected macrophages, indicating that *Salmonella* possesses active mechanisms to alter host cell signaling in intestinal epithelial cells which enhances PGE_2_ production (Resta-Lenert and Barrett, 2002; Uchiya and Nikai, 2004). *Salmonella*-induced PGE_2_ activates the protein kinase A (PKA) pathway and upregulates IL-10 production by macrophages, promoting an immunosuppressive phenotype and impaired killing ability. COX inhibition by indomethacin or SC-58125 restores the bactericidal properties of macrophages during *Salmonella* infection *in vitro* (Uchiya and Nikai, 2004). PGE_2_ production is also dependent upon the expression of *Salmonella* DNA adenine methylase (*dam*). *Salmonella dam* mutants are unable to promote COX-2 expression, leading to reduced PGE_2_ production in infected murine macrophages (Cristina Cerquetti et al., 2008). Along with the inability to elicit a strong PGE_2_ response, *dam* mutants are less cytotoxic to M cells, deficient in cell invasion (García-Del Portillo et al., 1999), and confer cross-protective *Salmonella* immunity in a mouse model (Heithoff et al., 2001).

During experimental salmonellosis with *S. typhimurium*, COX-2 expression and PGE_2_ concentrations in macrophages and dendritic cells within the mesenteric lymph nodes remain elevated 3 days after intragastric infection in the mouse model. At early stages of acute infection in the mouse model, COX-2 inhibition with celecoxib leads to an increase in bacterial loads in the mesenteric lymph nodes; however, at later stages of infection, COX-2 inhibition enhances host survival (Bowman and Bost, 2004). Thus, while PGE_2_ may have beneficial proinflammatory properties during acute *Salmonella* infection, prolonged exposure to PGE_2_ may be detrimental and promote an environment susceptible to chronic disease.

#### Escherichia coli

Enteropathogenic *E. coli* (EPEC) and enterohemorrhagic *E. coli* (EHEC) are Gram-negative bacteria that colonize the intestine and cause diarrheal disease. Both EPEC and EHEC induce PGE_2_ production by intestinal epithelial cells, however the most potent inducers of PGE_2_ are invasive strains such as *E. coli* O29:NM (Eckmann et al., 1997). The Type 3-secreted effector EspT is a guanine nucleotide exchange factor important for EPEC cellular invasion. EPEC strains expressing EspT promote increased COX-2 expression and PGE_2_ production by infected macrophages (Raymond et al., 2011). This suggests PGE_2_ increases in part through EspT expression and does not rely entirely on passive immune recognition of LPS or other Toll-like receptor (TLR) agonists, such as flagellin. This also suggests bacteria utilize active signaling mechanisms to exploit PGE_2_ for intracellular survival. High concentrations of *E. coli* LPS also induce PGE_2_ production by macrophages (Kurland and Bockman, 1978; Rosenstreich et al., 1978). *E. coli* LPS administered at 40 mg/kg is 100% fatal in the normal mouse model, yet COX-2^−/−^ mice demonstrate 100% survival at this dosage and are significantly protected against LPS doses as high as 100 mg/kg (Ejima et al., 2003). Accordingly, COX-2 inhibition may represent a therapeutic strategy in controlling infection with pathogenic *E. coli*.

#### Other enteric species

It is not surprising that additional enteric pathogens are able to elicit a PGE_2_ response upon infection. *Vibrio cholerae* is an enteric bacterial pathogen whose infection leads to acute watery diarrhea and an increase in PGE_2_ secretion in infected intestinal tissues. Specifically, jejunal fluids from patients presenting with acute cholera infection contain increased concentrations of PGE_2_ (Speelman et al., 1985). Both children and adults infected with *V. cholerae* O1 and *V. cholerae* O139 demonstrate significantly higher concentrations of PGE_2_ in stools when compared to healthy controls during the acute stages of infection. However, there is no significant difference in plasma PGE_2_ levels in these patients, suggesting the PGE_2_ response is restricted to the infected mucosa (Qadri et al., 2002). Cholera toxin (CT) also influences PGE_2_ production, as murine macrophages display enhanced PLA2 activity and PGE_2_ synthesis when stimulated with exogenous CT (Burch et al., 1988). Similarly, stimulation of isolated intestinal rabbit cells with CT leads to an increase in PGE_2_ concentrations (Peterson et al., 1994).

Other enteric bacterial pathogens demonstrate an ability to induce PGE_2_ production by infected cells. Both pediatric and adult patients presenting with acute shigellosis exhibit significantly higher concentrations of PGE_2_ in stool samples when compared to healthy controls (Raqib et al., 2000). Further studies must be conducted in order to determine the mechanisms by which enteric pathogens elicit PGE_2_ production in infected cells. Moreover, it will be necessary to determine how PGE_2_ concentrations affect both the host immune response and bacterial pathogenesis at various stages of enteric infection.

### Pulmonary infections

#### Mycobacteria

*Mycobacteria* are acid-fast bacilli that cause progressive or latent pulmonary disease after aerosol inhalation (Torrado et al., 2011). Several *Mycobacteria* species induce PGE_2_ production during infection. In the mouse model, *M. intracellulare* induces PGE_2_ synthesis, inhibiting the production of lymphokines in infected macrophages and suppressing an effective immune response (Edwards et al., 1986). *M. bovis* bacillus Calmette-Guerin (BCG) also enhances COX-2 expression and PGE_2_ production in a TLR2-dependent manner in infected macrophages *in vitro* and in a mouse model (Bansal et al., 2009). In particular, the presence of PGE_2_ has been noted in the sera and cerebrospinal fluid of tuberculosis patients (Bansal et al., 2009). Mice infected with *M. tuberculosis* demonstrate a 13-fold increase in lung PGE_2_ levels at 30 days post-infection compared to uninfected mice (Peres-Buzalaf et al., 2011). Granuloma formation, a hallmark of tuberculosis infection, is comprised of macrophages exhibiting high levels of COX-2 expression and PGE_2_ synthesis in the mouse model (Rangel Moreno et al., 2002). A gene encoding early secreted antigenic target protein 6 (ESAT-6), present in all pathogenic strains of *Mycobacterium*, induces COX-2 expression and PGE_2_ production in a TLR2-dependent manner in infected macrophages *in vitro* (A et al., 2012). Interestingly, the avirulent *M. tuberculosis* stain H37Ra was shown to promote macrophage PGE_2_ production leading to cellular apoptosis, while the virulent strain H37Rv induced significantly less PGE_2_ and caused macrophage necrosis (Chen et al., 2008; Divangahi et al., 2009). PGES^−/−^ macrophages are unable to control H37Rv replication and PGES^−/−^ mice demonstrate significantly higher bacterial burdens at 5 weeks post-infection with virulent *M. tuberculosis*, suggesting that PGE_2_ is necessary to control *M. tuberculosis* during the early stage of infection (Chen et al., 2008). Similar results were reported by Rangel Moreno et al. (2002) using wild type mice infected with H37Rv. COX-2, PGES, and PGE_2_ expression were low and relatively stable during the early phase of infection (up to 21 days), and COX-2 inhibition during early infection led to increased bacterial growth and immunopathology. In contrast, COX-2, PGES, and PGE_2_ expression increased during the chronic phase of infection (60–90 days), and inhibition of COX-2 led to increased iNOS expression with a concomitant reduction in lung bacterial load and granuloma size (Rangel Moreno et al., 2002). Clearly modulation of PGE_2_ can impact disease outcome during *M. tuberculosis* infection, and the consequences of PGE_2_ inhibition may differ between acute and chronic stages of tuberculosis infection. Therapeutic strategies targeting PGE_2_ may lead to alternative therapies in controlling *Mycobacterium* infection in the lung.

#### Streptococcus

Community-acquired pneumonia is one of the leading causes of death worldwide (Finch, 2001), and is most commonly caused by *S. pneumoniae* (Mandell et al., 2007). In patients suffering from acute pneumonia, COX-2 is expressed in alveolar epithelial cells (AECs). Similarly, AECs, alveolar macrophages, and vascular endothelial cells of human lung tissue *in vitro* exhibit time-dependent increases in both COX-2 expression and PGE_2_ production post-infection with *S. pneumoniae* (Szymanski et al., 2012). Streptococcal toxins also promote PGE_2_ production in immune cells. Particularly, pneumolysin produced by *S. pneumoniae* promotes the production of PGE_2_ in neutrophils and endothelial cells by inducing the expression of PLA2 (Rubins et al., 1994; Cockeran et al., 2001). Enhanced PGE_2_ production by neutrophils treated with pneumolysin inhibits an effective immune response by obstructing neutrophil activation and migration (Takenawa et al., 1986; Cockeran et al., 2001; Burelout et al., 2004, 2007). Inhibiting PGE_2_ production during *Streptococcus* infection enhances macrophage phagocytosis and generation of reactive oxygen species, aiding in bacterial clearance (Stables et al., 2010). PGE_2_ signaling post-*Streptococcus* infection relies on both EP2 and EP4 signaling (Aronoff et al., 2012; Szymanski et al., 2012). EP2^−/−^ murine alveolar macrophages demonstrate enhanced phagocytosis, intracellular killing, and increased generation of reactive oxygen *in vitro*, while EP2^−/−^ mice demonstrate improved bacterial clearance and survival post-infection with *S. pneumoniae*. Animal survival may be associated with a heightened production of pro-inflammatory cytokines, such as IL-12p40 (Aronoff et al., 2012). EP3 also plays a large role in PGE_2_ signaling post-infection with *Streptococcus* both *in vitro* and in a mouse model. EP3^−/−^ macrophages *in vitro* have enhanced phagocytic properties and bacterial killing mechanisms, such as nitric oxide production. EP3^−/−^ mice also exhibit greater levels of protection against *S. pneumoniae* when compared to wildtype mice. Specifically, EP3^−/−^ mice demonstrate heightened bacterial clearance in the lung by alveolar macrophages, with a decrease in infiltrating lung neutrophils and blood leukocytes (Aronoff et al., 2009). The immunosuppressive qualities of PGE_2_ have characteristically been attributed to EP2 and EP4 signaling, but EP3 signaling also contributes to increased production of PGE_2_ during pneumococcal infection.

Other species of *Streptococcus* induce an increase in PGE_2_ synthesis during pulmonary infection as well. Group B *Streptococcus* is a leading cause of neonatal sepsis and pneumonia, and infection with this bacterial pathogen leads to enhanced expression of COX-2 and increased concentrations of PGE_2_ in A549 human lung epithelial cells (Glibetic et al., 2001; Natarajan et al., 2007). *S. pyogenes*, a causative agent of pharyngitis, induces the expression of COX-2 and PGE_2_ synthesis in the macrophages of tissue biopsies from infected patients as well as in infected mice. Pharmacological inhibition of PGE_2_ synthesis by PKI (14–22) or genetic ablation of COX-2 expression promotes bacterial clearance and improves disease outcome in the mouse model (Goldmann et al., 2010).

#### Pseudomonas aeruginosa

*P. aeruginosa* is one of the most virulent opportunistic pathogens and is the leading cause of morbidity and mortality in cystic fibrosis patients (Sato et al., 2003; Sadikot et al., 2005). *P. aeruginosa* is also a common cause of hospital-acquired pneumonia (Sadikot et al., 2005). In a murine model of *P. aeruginosa* infection, overproduction of PGE_2_ in the lung diminishes phagocytosis and TNF-α production by alveolar macrophages (Ballinger et al., 2006; Hubbard et al., 2010). The inhibitory effects of PGE_2_ appear to partially signal through EP2, as EP2^−/−^ mice demonstrate decreased bacterial loads post-infection (Sadikot et al., 2007). *P. aeruginosa* induces cPLA2 activity within infected A549 epithelial cells in an ERK 1/2-dependent manner to trigger a four-fold increase in PGE_2_ production, which can be suppressed with the use of a specific cPLA2 inhibitor (Hurley et al., 2011). COX-2-deficient mice display enhanced bacterial clearance post-infection when compared to wildtype control mice. Recruitment of inflammatory cells in COX-2-deficient mice does not differ from those of control mice post-infection, suggesting bacterial clearance is associated with impaired effector functions of immune cells (Sadikot et al., 2007). Inhibition of COX-2 expression also decreases the severity of *P. aeruginosa* infection and increases survival rates in mice (Saliba et al., 2005; Sadikot et al., 2007). Murine bone marrow-derived macrophages treated with the selective COX-2 inhibitor NS-398 prior to infection with *P. aeruginosa* have lower concentrations of PGE_2_ and show an increase in superoxide production post-infection when compared to mock-treated controls (Sadikot et al., 2005).

#### Other pulmonary species

*Burkholderia pseudomallei* is a facultative intracellular Gram- negative bacillus that causes a fatal disease known as melioidosis. Patients acquire the infection through different routes and can present with a wide range of clinical symptoms including debilitating pneumonia and septic shock (Cheng and Currie, 2005). Recent work from our laboratory has demonstrated that PGE_2_ plays a critical role in the pathogenesis of *B. pseudomallei* infection in mice (Asakrah et al., 2013). PGE_2_ promotes *B. pseudomallei* intracellular survival through the activation of arginase 2 which competes with inducible nitric oxide synthase for the substrate, L-arginine, thereby limiting nitric oxide production. This process is antagonized by blocking PGE_2_ synthesis with a selective COX-2 inhibitor, NS398 (Asakrah et al., 2013). Treatment of bone marrow-derived macrophages with NS398 reduces endogenous PGE_2_ production and intracellular survival of *B. pseudomallei*. Conversely, addition of exogenous PGE_2_ to NS398-treated macrophages restores *B. pseudomallei* survival. Administration of NS-398 or Celecoxib significantly enhances mouse survival from lethal pulmonary infection with *B. pseudomallei* (Asakrah et al., 2013).

*Burkholderia cepacia* is a Gram-negative bacterium that causes fatal lung infections in cystic fibrosis patients. Approximately 20% of infected patients have severe pulmonary epithelial deterioration that can lead to death within a matter of weeks (Isles et al., 1984). In human lung epithelial cells, *B. cepacia* promotes enhanced PGE_2_ synthesis, possibly increasing the severity of disease in immunocompromised individuals (Fink et al., 2003). *Bordatella pertussis* infections result in a severe pulmonary illness known as pertussis or “whooping cough.” Pertussis toxin (PT) stimulates an increase in PGE_2_ production in infected murine macrophages *in vitro* (Burch et al., 1988; Schulze-Specking et al., 1991). Further research is warranted to identify the mechanisms behind which various pulmonary pathogens modulate PGE_2_ responses in the lung in order to aid infection.

### Active induction of PGE_2_

When inactivated, many bacteria are unable to elicit a strong PGE_2_ response by host cells. For example, when compared to live bacteria, UV-irradiated *S. typhimurium* are unable to induce COX-2 expression in infected macrophages, suggesting that *Salmonella* uses active mechanisms to alter gene expression in infected tissues for the production of PGE_2_ (Bowman and Bost, 2004). Similarly, UV-irradiated *S. aureus* are unable to promote PGE_2_ biosynthesis in infected osteoblasts (Somayaji et al., 2008). Both live and gamma-irradiated *M. avium* induce PGE_2_ production in infected human peripheral blood monocyte-derived macrophages, yet gamma-irradiated *M. avium* induce significantly lower concentrations of PGE_2_ (Rastogi et al., 1992). Heat inactivation of *B. pseudomallei* also led to a significant reduction in COX-2 expression and PGE_2_ production by murine macrophages (Asakrah et al., 2013). The reduced ability of inactivated bacteria to elicit a strong PGE_2_ response during infection suggests these bacteria have evolved active mechanisms to alter host cell signaling to promote PGE_2_ synthesis that may aid infection.

Type three secretion systems (T3SS) are important bacterial secretion systems, some of which stimulate PGE_2_ production during bacterial pathogenesis (Sato et al., 2003; Saliba et al., 2005; Sadikot et al., 2007; Raymond et al., 2011). ExoU is a T3SS effector molecule associated with *P. aeruginosa* infections which lead to nosocomial pneumonia and bacteremia (Berthelot et al., 2003; Schulert et al., 2003). This cytotoxin possesses phospholipase activity and induces rapid AA release from the cell wall and enhances PGE_2_ production during the infection of human epithelial cells (Sato and Frank, 2004; Saliba et al., 2005; Sadikot et al., 2007). Mice infected with ExoU-deficient *P. aeruginosa* have a significant decrease in COX-2 expression and diminished PGE_2_ production in the lung and a lower bacterial load in infected tissue, indicating that the secretion of this effector molecule aids in establishing infection (Saliba et al., 2005; Sadikot et al., 2007). *E. coli* also utilizes a T3SS effector molecule, EspT, to elicit a PGE_2_ response in infected macrophages (Raymond et al., 2011). Taken together, these studies highlight a conserved mechanism among bacterial T3SSs that induce PGE_2_ production during infection, and elucidation of these effectors may identify new therapeutic targets.

## PGE_2_ as a potential therapeutic target during bacterial infection

### COX-2 inhibition

Since PGE_2_ production has inhibitory effects on immune cells, particularly those involved in innate immune responses, inhibition of PGE_2_ may benefit the host during bacterial infection (Goto et al., 1983; Kunkel et al., 1986; Phipps et al., 1991; Strassmann et al., 1994; Kaliński et al., 1997; Harris et al., 2002). In support of this, mice deficient in COX-2 demonstrate enhanced survival post-infection with several bacterial pathogens. For example, COX-2^−/−^ mice exposed intraperitoneally to high doses of *E. coli* endotoxin exhibit increased survival compared to wildtype mice (Ejima et al., 2003). COX-2-deficient mice also demonstrate greater survival rates and exhibit lower bacterial loads in the liver and spleen after intravenous infection with *S. pyogenes* (Bowman and Bost, 2004). When compared to wildtype mice, COX-2^−/−^ mice exhibit increased bacterial clearance and enhanced survival at 6 days post-intratracheal infection with *P. aeruginosa* (Sadikot et al., 2007).

COX inhibitors, which are already widely used in the human population for the relief of pain and inflammation, block the production of PGE_2_ and other prostaglandins and may offer therapeutic benefit during bacterial infections. For example, non-selective COX inhibitors such as ibuprofen and indomethacin, significantly reduce the bacterial load and PGE_2_ production in the bronchoalveolar lavage (BAL) after intratracheal *P. aeruginosa* infection in mice (Saliba et al., 2005). COX-2 inhibition by NS-398 also significantly improves mouse survival post-intratracheal infection with lethal doses of *P. aeruginosa* (Sadikot et al., 2007). Moreover, NS398 administered post-exposure to mice infected with *B. pseudomallei* significantly reduces lung PGE_2_ levels and enhances animal survival (Asakrah et al., 2013). COX-2 inhibition results in higher bacterial loads during acute *S. typhimurium* and *M. tuberculosis* infection in mouse models, however administration of a COX-2 inhibitor during chronic infection with *S. typhimurium* or *M. tuberculosis* improves host protection (Rangel Moreno et al., 2002; Bowman and Bost, 2004). Similarly, Celecoxib treatment reduces lung levels of PGE_2_ and enhances the 60-day survival of *M. tuberculosis*-infected mice (Peres-Buzalaf et al., 2011). Because COX-2 inhibition impairs the production of prostaglandins in addition to PGE_2_, it is important to consider the potential contribution of other prostaglandins in such studies. Furthermore, additional studies in highly relevant animal models are needed to determine the therapeutic efficacy of COX inhibitors against mucosal bacterial infections.

### Receptor inhibition

Specific targeting of one or more PGE_2_ receptors may also hold therapeutic promise. EP2 is a major receptor responsible for the immunosuppressive activities of PGE_2_ signaling (Fujino et al., 2005). EP2^−/−^ alveolar macrophages exhibit improved phagocytosis, increased production of reactive oxygen intermediates and pro-inflammatory cytokines, such as TNF-α and MIP-2, and enhanced killing of *P. aeruginosa* (Aronoff et al., 2012). Impaired EP2 signaling improves disease outcome in *P. aeruginosa*-infected mice, as EP2-deficient mice show enhanced survival and bacterial clearance correlated with enhanced neutrophil migration and IL-12 production in the lung (Sadikot et al., 2007; Aronoff et al., 2012). Inhibition of EP3 may also be beneficial in controlling bacterial infections. EP3-deficient alveolar macrophages demonstrate increased phagocytic activity and nitric oxide production, and enhanced bacterial killing during *S. pneumoniae* infection. EP3^−/−^ mice exhibit greater bacterial clearance and higher survival post-intraperitoneal infection (Aronoff et al., 2009). Specific EP inhibitors or antagonists may aid in therapeutically controlling microbial infection and require further study.

## Conclusions and future directions

PGE_2_ is an important lipid mediator that regulates inflammation and immune responses during infection (Phipps et al., 1991; Yu and Chadee, 1998; Harris et al., 2002; Nagamatsu and Schust, 2010). Four principle PGE_2_ receptors respond to varying concentrations of PGE_2_ in order to elicit dynamic downstream signaling events during immune responses. It is increasingly evident that PGE_2_ biosynthesis and its inhibitory actions on innate immune defenses can impact bacterial pathogenesis and disease outcome. For infected macrophages, PGE_2_ production correlates with diminished phagocytosis, nitric oxide production, and intracellular killing (Marotta et al., 1992; Aronoff et al., 2004; Hubbard et al., 2010), and promotes an immunosuppressive cytokine profile (Kunkel et al., 1986, 1988; Huang et al., 1998; Stolina et al., 2000; Liu et al., 2012). Neutrophil and NK cell activation, migration, and aggregation are inhibited by PGE_2_ (Bankhurst, 1982; Goto et al., 1983; Takenawa et al., 1986; Joshi et al., 2001; Burelout et al., 2004, 2007; Walker and Rotondo, 2004). PGE_2_ shifts the immune response away from a Th1 response and toward a Th2 response by promoting the production of anti-inflammatory cytokines and modulating the interactions between DCs and other immune cells (Kaliński et al., 1997, 1998; Gustafsson et al., 2008; Obermajer et al., 2011). The conserved ability of many bacterial pathogens to promote PGE_2_ responses during infection suggests a common signaling mechanism to deter protective pro-inflammatory immune responses. Inhibition of PGE_2_ production and signaling during infection may represent a therapeutic alternative to treat certain bacterial infections. Further study of the immunosuppressive effects of PGE_2_ on innate immunity will lead to a better understanding of potential therapeutic targets within the PGE_2_ pathway.

### Conflict of interest statement

The authors declare that the research was conducted in the absence of any commercial or financial relationships that could be construed as a potential conflict of interest.
